# The Action of Iodide of Potash

**Published:** 1890-04

**Authors:** L. Trasbot


### CASES, SELECTIONS, TRANSLATIONS.

### THE ACTION OF IODIDE OF POTASH.

By Prof. L. Trasbot.

More than ten years ago, Professor Trasbot, of Alfort, commenced the study of the action of iodide of potash in animals, and he has obtained most remarkable results from its use in the treatment of certain affections of the lungs, of the bronchi and of the heart.

He has determined its physiological to be: First, augmentation of the force and frequency of the pulse, tension of the arteries, slight acceleration of the respiration, and rapid engorging of the conjunctiva and respiratory mucous membrane; then, reduction of the number of pulsations with fullness of the pulse and softness of the arteries, slowing of the respiration, salivation, abundant secretion of tears, and muco-serous discharge from the nasal membranes.

But iodide of potash also exercises, like digitalis, with which it has some physiological properties in common, an antithermic action, decidedly marked. This action, which is manifested as soon as the vaso-dilatation commences, lowers the temperature almost one degree in animals in health. Further, if the medication is continued for several days, it determines an emaciation—a notable loss in weight in the animal.

"These complex effects," says Mr. Trasbot, "inspired me with the idea of having recourse to this agent for combatting pulmonary congestion, pneumonia, bronchitis, attacks of heaves and chronic affections of the heart, especially in the horse. The results which I have obtained under diverse circumstances are most remarkable, and prove that the iodide of potash, without constituting, be it understood, a specific, can render the greatest service in the treatment of these diverse diseases

"Frequently I have had occasion to administer it in pulmonary congestion to second the effects of bleeding and of revulsives, sometimes with digitalis, but also alone in some cases, and it has always appeared to me to exercise a powerful action. In several hours after its administration in small doses, 12 to 16 grammes (ʒiij to iv) in the day the pulse is seen to slow and become more full, the respirations diminish in number and become more ample and all menace of asphyxia disappears.

"It is not less useful in the treatment of acute pneumonia. I had given it at first on account of its action on the lymphatic glands, with the object of reducing as much as possible the swelling of those of the bronchi, the pressure of which on the left recurrent laryngeal nerve is the cause of persistent roaring so common after pneumonia and bronchitis, and so harmful to the usefulness of the horse and consequently to his value. I can say here that, from this special point of view, iodide of potash is absolutely precious. A very large number of animals to which I have given it have recovered without being roarers. For me, as I showed in the memoir which I published on distemper, there is no longer a doubt of its preventive influence in this trouble. But I have also quickly recognized its really curative action in pneumonia.

"Therefore, for a long time, I have never missed combining iodide of potash in the treatment of pneumonia; not to the exclusion of the other classical remedies (bleeding at first in certain cases, revulsives, digitalis and antimony) but as an adjuvant of great utility.

"It has an equally remarkable efficiency in the treatment of bronchitis. Frequently I have employed it alone in treating this affection. In a very short time it lowers the fever and provokes a moist bronchial secretion, which rapidly brings on a resolution of the disease. I ought, however, to state that I generally combine it with Kermes mineral."

It has always, over the antimonials and especially over tartar emetic, which, even in small doses, often irritates the intestines, the considerable advantage of producing no evil effects in this way, but on the contrary it excites the appetite.

Its action is still more powerful in combatting rapid attacks of heaves, whether these attacks are due to emphysema alone, or to this and a chronic bronchitis combined, which is the most common condition; the two alterations often co-existing, it gives astounding results. As early as 1865, Plantin, veterinarian at

Marseilles, in the *Clinique Vétérinaire* of Leblanc, recommended the iodide of potash in the heaves and chronic bronchitis, as being a great deal more efficacious than arsenic.

Finally, it gives very advantageous results in nearly all the chronic affections of the heart, diseases for a long time unknown and yet extremely frequent in the horse, which is readily explained by the efforts of force which are required of this animal.

In all cases of chronic endocarditis it produces a notable amelioration in a few days. I have seen a large number of subjects rendered incapable of service, stopping during work, paying no attention to the whip, enfeebled and having lost their appetite, regain the appetite and the appearance of health in a few days. They are not cured radically, of course, but they seem to be so enough to be sold and resold, until the day when they go to the butcher[1] or the knacker.

There is one condition especially in which the iodide of potash produces astonishing effects, that is, the case of pulmonary œdema complicating dilatation of the heart, a very common condition in the horse, whether it exists alone, or with pulmonary emphysema and mitral induration, which is also frequent. Several times I have seen accidents of this sort, which threatened a quick termination to the life of the animal, succumb rapidly to an iodized medication. I should add, however, that usually, in these circumstances, I combined it with digitalis in moderate doses. But to prove the action of the iodide of potash, I have also given it alone, and I have obtained, somewhat less promptly but to a complete degree, the resolution of the trouble.

In short, it appears to me to be a precious remedy in a number of morbid conditions. H.

[1] It would be well if the United States markets offered a haven for horses, in good health except some chronic trouble which renders them unserviceable, and a cheap good food for the people.—Ed.]

